# Role of Otolaryngology in Monitoring the Progression of Myotonic Dystrophy: A Case Report

**DOI:** 10.7759/cureus.104732

**Published:** 2026-03-05

**Authors:** Varsha Pudi, Akash Shah

**Affiliations:** 1 Internal Medicine, University of Vermont, Larner College of Medicine, Burlington, USA; 2 Internal Medicine, Nuvance Health, Brookfield, USA

**Keywords:** ent - ear nose and throat, multi-disciplinary teams, myotonic dystrophy type 1, progressive dysphagia, voice changes

## Abstract

Myotonic dystrophy is a systemic condition with the involvement of the neuromuscular, cardiac, and gastrointestinal systems. Otolaryngology has an underrecognized yet critical role in the longitudinal management of myotonic dystrophy, given the impact on swallowing, voice, and airway management. This case is of a 42-year-old male with genetically confirmed myotonic dystrophy type one. The patient presented with complaints of dysphagia, voice changes, and hand weakness. Though this patient’s workup primarily focused on internal medicine and gastroenterology, these quality-of-life complaints highlight a realm where otolaryngology monitoring and evaluation can contribute to early recognition and management of aspiration risk, vocal dysfunction, airway management, and sleep apnea, ultimately improving quality of life. This shows the value of a multidisciplinary team, including otolaryngology, in promoting the overall health and quality-of-life goals of patients with myotonic dystrophy.

## Introduction

Myotonic dystrophy is a neuromuscular disorder that impacts skeletal and smooth muscle with widespread systemic involvement [1]. The condition, which includes several subtypes, affects millions of individuals worldwide [2]. In the most common form of myotonic dystrophy, cytosine-thymine-guanine (CTG) trinucleotide repeats in the DMPK gene lead to the accumulation of toxic aggregates, resulting in impaired muscle relaxation and progressive muscle weakness [2,3]. While certain cardiac and musculoskeletal complications are well-recognized, there are a number of otolaryngologic manifestations, such as dysphagia, airway compromise, and voice changes, which are underrecognized in the care of these patients [4]. As the condition progresses from the classic presentation of delayed handgrip release, patients experience further muscle wasting and myotonia, and eventually may develop issues relating to speech and language. Speech, language, and swallowing require coordinated muscle movements of the upper esophageal sphincter, pharynx, and suprahyoid muscles [5]. Progressive dysfunction of these muscles increases safety concerns such as aspiration. Patients may develop difficulty in speech production, fluency, and intelligibility in addition to hoarseness, hypernasality, and limitation of communication [5]. These complications can significantly impact patient safety and quality of life. This case discusses the potential role of otolaryngology in complementing multidisciplinary monitoring and management of patients with myotonic dystrophy.

## Case presentation

A male in his 40s with a past medical history of *Helicobacter pylori* (*H. pylori*), sleep apnea, elevated liver function tests (LFTs) secondary to fatty liver, and progressive myotonic dystrophy presented for his annual physical after several years of inconsistent continuity of care. As a teenager, the patient noticed that he began to experience difficulty with releasing his grip after a handshake. And over the next eight years, the patient began to develop worsening hand weakness and fine motor impairment. Following an extensive workup, including rheumatology, orthopedics, and neurology, the patient received a clinical diagnosis of myotonic dystrophy type 1. Genetic testing confirmed a diagnosis of myotonic dystrophy with 130 CTG repeats in the DMPK gene. The patient also reports percussion myotonia of the left thenar eminence.

**Figure 1:**
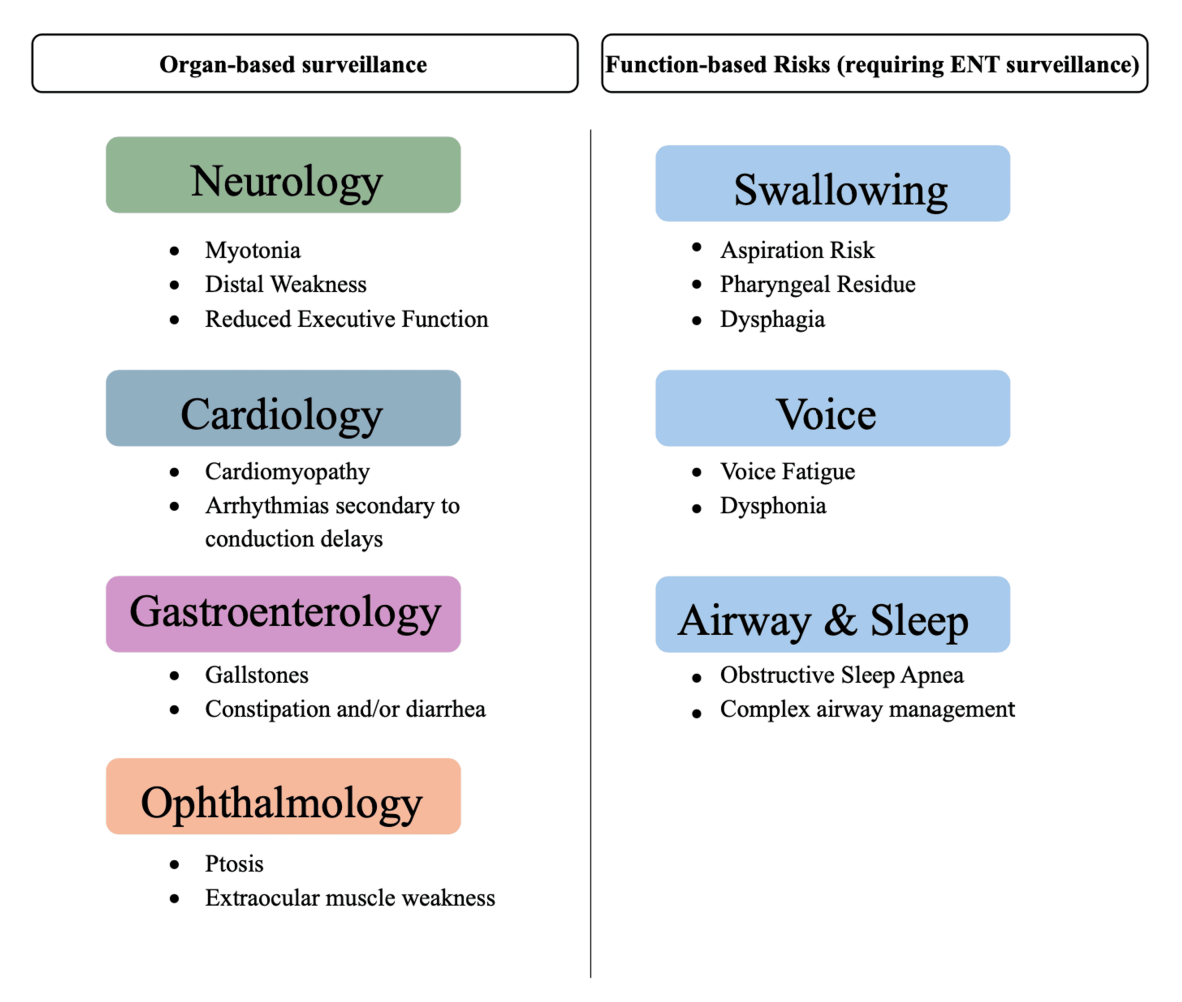
Function-based otolaryngologic risk domains in myotonic dystrophy

**Figure 2:**
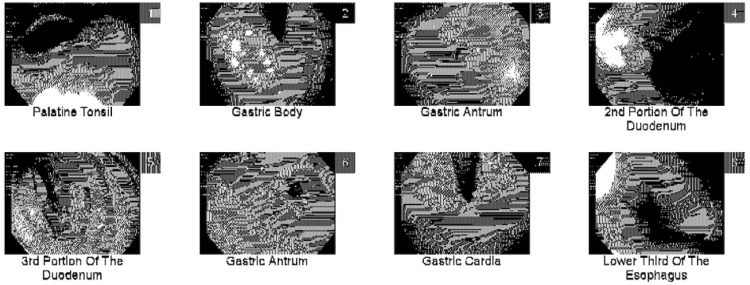
Upper endoscopy demonstrating a patulous esophagus with moderate to severe dysmotility, a Schatzki B ring, and an axial hiatal hernia without evidence of gastroesophageal reflux disease. Note: Image quality is limited.

Via a detailed chart review, the patient has a significant history of nocturnal heartburn and regurgitation, in addition to intermittent difficulty swallowing solids (Figure 1). The patient underwent an upper endoscopy, which revealed a patulous esophagus with moderate to severe dysmotility, a Schatzki B ring, and an axial hiatal hernia, without evidence of gastroesophageal reflux disease (Figure 2). During the upper endoscopy, the patient was also found to have *H. pylori *and was successfully treated with tetracycline, bismuth, metronidazole, and a proton pump inhibitor.

On physical exam, the patient had weakness in his distal lower extremities and hands and had convergence palsy. The patient also developed a softer voice as the interview progressed, which he states has been worsening.

## Discussion

Myotonic dystrophy is a systemic condition that impacts skeletal and smooth muscle, including but not limited to cardiomyopathy, myopathy, and myotonia. It is an autosomal dominant condition that is associated with CTG repeats. Patients with adult-onset myotonic dystrophy type 1 tend to carry more than 100 CTG repeats, and the expansive length tends to be predictive of clinical severity and age of onset [6]. Therefore, the patient, having 130 CTG repeats and symptoms that started at the age of 19, is consistent with the expected clinical progression of adult-onset myotonic dystrophy type 1. Although the Myotonic Dystrophy Foundation describes the importance of an interdisciplinary team to manage symptoms, notably otolaryngology is not explicitly listed as a core member of the team [7]. Given the functional swallowing, voice, and airway concerns involved in myotonic dystrophy, incorporation of otolaryngology in this team may complement existing care pathways and contribute to improved quality of life.

Otolaryngologists are in a unique position to monitor functions that are crucial to quality of life and overall safety in myotonic dystrophy. Oropharyngeal weakness can contribute to dysphagia and aspiration risk, which can be monitored by otolaryngologists. One of the patient’s major complaints involved intermittent difficulty swallowing solids. A study found that patients can be found with pharyngeal residue, which initially may not present as an issue; however, residue after swallowing larger volumes (20 mL) can present a four-fold risk for aspiration [8]. Several tools exist for formally assessing the patient’s swallowing function, such as videofluoroscopic swallow study or fiberoptic endoscopic evaluation, which may have provided a more comprehensive picture of the patient's oropharyngeal swallowing function and aspiration risk. The absence of such testing in this case highlights a potential gap in multidisciplinary evaluation of patients with myotonic dystrophy and the value of early otolaryngology involvement. Routine monitoring for these concerns can help prevent future risk of aspiration or help clinicians with providing recommendations for safety purposes. Studies have shown that high-resolution pharyngeal manometry that measures swallowing pressures at different pharyngeal locations can reveal a marked decline in pharyngeal swallowing motor function, even if patients do not complain of subjective symptoms, though this testing was not done with our patient [9]. This monitoring and potential intervention can highlight value in patients with minimal symptoms.

One of the patient’s complaints involved changes in his voice after extensive periods of talking. This is associated with muscle weakness in terms of the tongue being unable to rise to the alveolar ridge, the inability of vocal folds to contact one another, or the inability of the velum to reach the back wall of the pharynx at a sufficient level to support speech production [10]. Otolaryngology can play a critical role in this aspect of quality of life for patients with myotonic dystrophy, with early identification of dysarthria to allow for timely interventions.

In addition to concerns for dysphagia and speech, airway management is also an area where otolaryngology services can be effective. Patients with myopathies, including myotonic dystrophy, carry the risk of prolonged respiratory depression, especially with anesthesia [11]. The conjunction of otolaryngology with anesthesia can anticipate airway challenges and improve overall anesthesia safety in myotonic dystrophy patients. Additionally, studies have found that sleep apnea may be common among patients with myotonic dystrophy [12], highlighting another area where otolaryngology can identify these concerns early and reduce associated morbidity.

While the management of myotonic dystrophy is likely primarily in the realm of neurology and cardiology, otolaryngology can play a unique role in terms of monitoring and management of quality of life and safety. Early recognition of ENT-associated complications can help improve the quality of life of patients with myotonic dystrophy.

## Conclusions

This case highlights the critical, yet underrecognized role otolaryngology has in the treatment and management of patients with myotonic dystrophy. Increased multidisciplinary care for patients with myotonic dystrophy is essential for comprehensive care. Routine otolaryngologic monitoring assesses for and manages dysphagia, aspiration risk, sleep apnea, and voice change concerns, allowing for timely interventions that improve safety, optimize functional outcomes, and improve quality of life for myotonic dystrophy patients.
